# Extracellular vesicles from prostate tumors reshape the pre-metastatic bone environment in an mTOR/RAB1A-dependent manner

**DOI:** 10.3389/fimmu.2025.1605494

**Published:** 2025-09-19

**Authors:** Tingting Lv, Yawen Guo, Yuehua Zhang, Jing Cao, Xing Li, Dehua Wang, Xiaokuan Zhang, Dongwei He, Xiaojin Guo, Chunwang Yang, Zhiyu Wang

**Affiliations:** Department of Immuno-Oncology, The Fourth Hospital of Hebei Medical University, Shijiazhuang, Hebei, China

**Keywords:** B lymphocytes, extracellular vesicles, pre-metastatic niche, prostate cancer, Rab1A

## Abstract

**Background:**

Bone is the most common metastatic site in prostate cancer (PCa) patients and serves as a key contributing factor to the poor prognosis observed in advanced-stage patients. Mammalian target of rapamycin (mTOR) inhibition has limited clinical efficacy, potentially due to pathway complexity. Prior to the colonization by tumor cells, primary PCa cells actively remodel the bone microenvironment through the secretion of mediators including extracellular vesicles (EVs). The objective of this research is to investigate the regulatory mechanisms of EV biogenesis and the effects of EVs on the bone pre-metastatic niche (PMN), offering a novel therapeutic strategy against bone metastasis.

**Methods:**

PCa cell lines were employed to detect mTOR and Ras-related protein Rab-1A (RAB1A) protein expression levels via Western blotting (WB). Functional assays (invasion and proliferation) were used to validate the impact of RAB1A expression on biological behavior. The biological characteristics of EVs were characterized using WB, nanoparticle tracking analysis, and transmission electron microscopy. Bone marrow cell subpopulation alterations were analyzed based on the GSE143791 single-cell dataset. Cells and animal models were treated with EVs to assess their effects on the bone marrow microenvironment, survival time, and bone metastatic burden. Finally, peripheral blood routine parameters were compared in patients with or without bone metastasis.

**Results:**

Utilizing PCa cell lines, we demonstrated that mTOR activation inhibits the ubiquitination activity of the oncogenic factor RAB1A, thereby stabilizing its expression. The EVs derived from tumor promoted bone immunosuppression via B-cell dysfunction and myeloid cell expansion, highlighting their role in PMN formation. In RAB1A-overexpressing PCa animal models, GW4869-mediated inhibition of EV secretion prolonged mice survival, ameliorated bone marrow abnormalities, enhanced B-cell activation capacity, and reduced regulatory B-cell proportions.

**Conclusions:**

Our findings elucidated the detailed mechanism by which mTOR/RAB1A regulates EV secretion, providing new insight into cellular changes involved in PMN formation and a theoretical basis for the inhibition of the PMN in the development of targeted therapies for PCa. RAB1A represents a therapeutic target to reverse tEV-mediated immunosuppression, while peripheral B-cell dynamics provide diagnostic biomarkers for early metastasis detection.

## 1 Introduction

Based on the global cancer statistics from the American Cancer Society journal *CA: A Cancer Journal for Clinicians*, prostate cancer (PCa) ranks as the leading malignancy of the male genitourinary system, accounting for 29% of new cancer cases among men worldwide (1, 2). The progression of PCa to castration-resistant prostate cancer (CRPC) is frequently driven by somatic alterations in the phosphoinositide 3-kinase (PI3K)/protein kinase B (AKT)/mammalian target of rapamycin (mTOR) pathway, indicating that therapies targeting this pathway could improve survival outcomes and therapeutic efficacy. However, mTOR blockade in CRPC results in limited effectiveness. In a phase 2 clinical trial (NCT00629525), CRPC patients treated with the mTOR inhibitor everolimus showed neither a decline in prostate-specific antigen levels nor objective clinical responses (3). This limited efficacy may stem from the inherent complexity and compensatory activation mechanisms within the mTOR signaling pathway (4). Thus, to explore precise therapeutic targets and enhance therapeutic strategies, we further analyzed the mTOR signaling pathway and its downstream proteins in PCa.

Bone metastasis is the most common site of metastasis in PCa patients (2). Apart from prostatectomy and androgen deprivation treatment, immunotherapy has failed to achieve the anticipated therapeutic benefits for PCa patients with bone metastasis (5). Mechanistically, prior to the occurrence of bone metastases, primary tumors can remotely distort bone marrow cell activity in response to pathological changes, reshaping the microenvironment of the pre-metastatic niche (PMN) to assist in the colonization and proliferation of circulating tumor cells (CTCs) (6). Extracellular vesicles (EVs) are small, highly heterogeneous membranous particles derived from various cell types, while RAS-related protein (RAB) GTPases act as regulatory switches to control EV trafficking and secretion (7). Numerous reports have described that tumor-derived EVs (tEVs) drive immunosuppressive PMN formation through multifaceted mechanisms, such as inactivation of the anti-tumor activities of natural killer (NK) cells and T cells, induction of myeloid-derived suppressor cell expansion and macrophage polarization (8, 9). As a common cell type in the bone marrow, B cells develop through defined stages (pre-pro-B, pro-B/pre-B, immature B) before migrating to secondary lymphoid organs for further maturation (10). Recent studies indicate that tumors can reprogram B cells to blunt immune responses and induce metastasis-supporting regulatory B cells to promote metastasis (11, 12). However, the impact of tEVs on B cell development and the role of B cells in tumor immunosuppression remain less understood. Thus, the tEV-mediated induction in B-cell development need intensive research, particularly regarding their specific contributions to the regulation of immunosuppression by the PMN.

The mTOR signaling pathway critically regulates the EV biogenesis and cargo loading (13, 14). Hyperactivation of mTORC1 in cancer cells enhances EV secretion, which in turn delivers oncogenic miRNAs (e.g., miR-21) and phosphorylated signaling proteins to recipient cells, indicating the complexity of the mTOR signaling pathway (15). According to the study by Thomas, Ras-related protein Rab-1A (RAB1A) overexpression positively correlates with mTOR activation and rapamycin sensitivity in colorectal cancer cell lines (16). Thus, we hypothesized that these studies on the regulatory mechanisms of mTOR and RAB1A in PCa would address the dilemma of the apparently low clinical utility of a single mTOR inhibitor in PCa patients.

Herein, we discover that inhibiting the mTOR/RAB1A axis suppresses EV secretion, thereby inhibiting the formation of the pre-metastatic immunosuppressive microenvironment. Our results further support that tEVs disrupt normal bone marrow cell development and induce an imbalance between B lymphocytes and myeloid cells, indicating that RAB1A may serve as a novel therapeutic target for PCa treatment.

## 2 Material and methods

### 2.1 Animals

Wild-type male C57BL/6 mice and nude mice at 6–8 weeks of age were purchased from Vital River and kept in a specific pathogen-free facility at constant temperature and humidity for 1 week prior to tumor cell inoculation or tail vein injection of tEVs. All animal experiments in this study were conducted in accordance with animal welfare law and institutional guidelines with the approval of the Institutional Animal Care and Use Committees at the Fourth Hospital of Hebei Medical University (Approval number: 2023212). Bone marrow cells were flushed out of the femur and tibia using phosphate-buffered saline (PBS) containing 1% fetal bovine serum (FBS, VivaCell, C04001-050X10) and a 27G syringe needle. Each batch of isolated primary cells was pooled from three mice. CD19^+^ B cells were isolated with a Mouse CD19^+^ B Cell Isolation Kit (480002, BioLegend) and the purity of magnetic-activated cell sorting (MACS)-purified CD19^+^ cells was > 95% (**Supplementary Figure S6A**).

### 2.2 Cell culture

PCa cell lines (human DU145 and PC-3, and murine RM-1 (Pricella) were cultured in Ham’s F12K (BasalMedia, L450KJ), MEM (Gibco) and RPMI 1640 (Gibco), respectively, each supplemented with 10% FBS and 1% penicillin-streptomycin solution. Bone marrow cells and MACS-purified CD19^+^ B cells were cultured in 1640 supplemented with 10% FBS, 1% penicillin-streptomycin solution, 2-Mercaptoethanol (50 µM). The cell culture medium used for tEVs isolation was formulated with 10% exosome-free FBS and 1% penicillin-streptomycin solution. Plasmid construction and lentiviral packaging were mentioned as before (17). shRNA-1 targeting RAB1A (human): 5-GGAAACCAGTGCTAAGAATGC-3, shRAB1A-2 targeting RAB1A (human): 5- CTTCTTAGGTTTGCAGATGAT -3. shRNA-1 targeting RAB1A (mus): 5- GGAGTCCTTCAATAACGTTAA-3, shRAB1A-2 targeting RAB1A (mus): 5-GCACAATTGGTGTGGATTTCA -3. The specific plasmid overexpressing RAB1A and luciferase lentivirus were purchased from Genechem (Shanghai). All cell lines were cultured in a humidified incubator with 5% CO2 at 37°C.

### 2.3 EV isolation

To mitigate the effects of apoptotic cells on the purity of EVs, it was advisable to collect the cell supernatant when the cells achieved approximately 80%. Cell culture medium containing EVs was pre-cleared first by a speed centrifugation step (3,000×g for 15 min) to remove debris and dead cells. Then, remaining larger particles were removed with a 0.45-µm filter. The culture medium was concentrated using 10-kDa MWCO Amicon Ultra-15 spin-filters (UFC901024, Millipore). The concentrated cell culture supernatant was utilized for the purification of EVs according to the exoEasy Maxi kit (76064, Qiagen) protocol.

### 2.4 RNA extraction and quantitative reverse transcription PCR (qRT-PCR)

Total RNA was extracted from the cultured cells using TriQuick Reagent (Solarbio, Beijing, China) and measured with a ND100 spectrophotometer (Nanodrop, Wilmington, DE, USA) according to a standard protocol. cDNA synthesis was conducted using PrimeScript™ RT reagent Kit with gDNA Eraser (Takara, Japan). The gene expression was quantified with qRT-PCR using SYBR Green PCR master mix (Yeasen, Shanghai, China). The relative quantitative analysis of genes were calculated by the 2-ΔΔCt method normalized against β-actin, an internal control gene. All primer sequences in RT-qPCR were provided in **Supplementary Table 1**.

### 2.5 Western blotting and co-immunoprecipitation

Cell samples were lysed in lysis buffer supplemented with protease inhibitors for 30 min. After centrifugation at 20,000×g for 10 min at 4 °C, the protein concentration of the supernatant was determined using a BCA Protein Assay Kit. Equal amounts of protein were separated by 8-12% SDS-PAGE and transferred onto PVDF membranes. The membranes were incubated overnight at 4°C with primary antibodies. The antibodies against β-actin (AC026), P70S6K1 (A2190), and phospho-P70S6K1 (AP0564) were from Abclonal; against mTOR (66888-1), RAB1A (11671-AP), GAPDH (60004-1-Ig), mTOR (66888-1), and RAB1A (11671-AP) were from Proteintech; against RAB27B (HA723666), RAB7 (ET1611-96), ALIX (ET-1705-74), TSG101 (ET1701-59), and calnexin (ER1803-42) were from Huabio; and against CD9 (380441) was from Zenbio. After washing, membranes were probed with species-matched secondary antibodies (goat anti-mouse: A0216, Beyotime; goat anti-rabbit: A0208, Beyotime). For Co-IP, antibodies against mTOR and RAB1A were mixed with magnetic beads were incubated with protein samples at 4°C. Subsequently, proteins were separated from the magnetic beads and detected by WB as previously described.

### 2.6 Single-cell data quality control

Single-cell RNA sequencing data were processed using R and standard pipelines. Raw counts were imported via Seurat’s Read10X function and stored as a dgCMatrix (18). Samples were merged into a single object, and cell identifiers were standardized with RenameCells. Doublets were detected and removed using Scrublet (19). Low-quality cells were filtered by excluding genes detected in fewer than 200 cells and removing cells containing <500 or >4,000 genes per cell. Gene expression was normalized via the LogNormalize method with a scale factor of 10,000. The FindVariableFeatures function was used to identify the top 2,000 variable genes for downstream analysis. Technical noise (UMI counts, mitochondrial content) was regressed out using ScaleData. Dimensionality reduction was performed via PCA (30 principal components), followed by batch correction using Harmony. Cells were visualized using UMAP. Clustering was conducted by constructing a shared nearest-neighbor graph with the Louvain algorithm (resolution tested: 0.1-1.0; optimal resolution =0.6, validated via clustree). Differential gene expression analysis was performed using FindAllMarkers, followed by marker-based annotation with the Cell Taxonomy database (https://ngdc.cncb.ac.cn/celltaxonomy/) and literature references to assign cell identities.

### 2.7 Pseudotime analysis

For pseudotime trajectory analysis, Seurat objects were converted to Monocle2 CellDataSet format using count matrices from the RNA assay. The conversion process involved creating AnnotatedDataFrame objects for both phenotypic and feature data, followed by establishing a CellDataSet with the negative binomial distribution family, appropriate for sparse count matrices. Size factors and dispersions were estimated using estimateSizeFactors() and estimateDispersions() functions, respectively, and expressed genes were detected with a minimum expression threshold of 3. Highly variable genes for trajectory inference were selected based on dispersion analysis using dispersionTable(), where genes with mean expression ≥ 0.1 and empirical dispersion ≥ fitted dispersion were retained as ordering genes via setOrderingFilter(). Dimensionality reduction was performed using the Discriminative Dimensionality Reduction via Learning a Tree (DDRTree) algorithm with reduceDimension() function, projecting cells into a two-dimensional space with maximum components set to 2. The DDRTree method constructs a tree-like trajectory structure that captures both linear and branching developmental paths while preserving the global structure of cell state transitions. Finally, cells were ordered along the inferred trajectory using orderCells() function, which assigns pseudotime values representing the progress of each cell along the developmental trajectory and identifies distinct cell states based on the tree structure. This approach enables the identification of transitional cell states located at branching points and the characterization of gene expression dynamics throughout the developmental process.

### 2.8 Characterization of tEVs

The expression of TSG101, CD9, calnexin, ALIX, and GAPDH was evaluated by WB. The size distribution and concentration of tEVs were determined using ZetaView (version 8.05.14 SP7). Each EV sample was injected and measured three times. Transmission electron microscopy (TEM) was employed to assess the size and morphology of EV particles using a HITACHI H-7650 instrument (Tokyo, Japan) following a previously described method (20).

### 2.9 Heat inactivation of tEVs

As described by Doste R. Mamand, tEVs were subjected to heat inactivation. The heat inactivation process involved using a heating block to incubate the tEVs at either 100°C for 10min (*in vivo* studies) or 56°C (*in vitro* studies) (21).

### 2.10 Cell uptake experiment

EVs derived from RM-1 were labeled using a 10 µM PKH26 staining solution (HY-D1451, MCE) according to the manufacturer guidelines, 2×10^9^ PKH26-labelled tEVs were injected through tail vein for 4 hours. Bone marrow cell uptake was analyzed by flow cytometry.

### 2.11 Tumor induction and tEVs treatment in mice

For tumor induction, male C57BL/6 mice were either non-injected or injected with 1 × 10^5^ RM-1 cells suspended in 100 µL PBS (n = 6) via the left cardiac ventricle (LCV). Bone metastases were monitored through serial body weight measurements, lameness assessment, and hematoxylin-eosin (HE) staining of femoral and tibial tissues (22, 23).

Male C57BL/6 mice received tail vein injections every 3 days for 2 weeks with PBS/1 × 10^9^ heat-inactivated tEV particles (100°C, 10 min)/1 × 10^9^ tEV particles (n=3).

Male C57BL/6 mice were randomly divided into three groups (n=6 per group): *Control Group* (100µL PBS administered via tail vein injection prior to intracardiac PBS injection), *PCa Group* (100µL PBS administered via tail vein injection prior to intracardiac RM-1 cell injection), *tEVs-treated PCa Group* (1 × 10^9^ tEV particles administered via tail vein injection prior to intracardiac RM-1 cell injection). Mice were injected through tail vein every 3 days for 2 weeks. Bone metastases were visualized by using a small-animal *in vivo* optical imaging system (IVIS).

Male C57BL/6 mice injected with RM-1 were randomly divided into five groups (n=6 per group): *PCa Group* (received daily intraperitoneal (i.p.) injections of PBS); *RAB1A Group* (received an LCV injection of RM-1 cells overexpressing RAB1A (RM-1 RAB1A-OE) and received i.p. injections of PBS daily); *GW4869 Group* (i.p. injections of GW4869 daily, HY-19363, MedChemExpress) at a dose of 2.5 mg/kg); *RAB1A+GW4869 Group* (received an LCV injection of RM-1 RAB1A-OE cells and i.p. injections of GW4869 at a dose of 2.5 mg/kg daily); and *tEVs Group* (i.p. injections of tEVs at a concentration of 1 × 10^9^ particles, every 3 days). A PBS-injected group of the same strain served as the *Control Group*.

### 2.12 Flow cytometric analysis

Bone marrow cells and peripheral blood (PB) cells were treated with red blood cell lysis buffer in the dark and on ice. Samples were stained with different combinations of anti-mouse flow cytometry antibodies according to manufacturer’s instructions. 7-aminoactinomycin D was used to immediately exclude dead cells. The samples were further evaluated utilizing flow cytometry and data were analyzed with FlowJo Software (Version 10.8.1). Detailed information of flow cytometry antibodies used in this study is listed in **Supplementary Table S2**.

### 2.13 Multiplex secretome analysis

Blood samples were collected into vacuum tubes containing EDTA. After thorough mixing for 10–20 mins, centrifuge at 1000 × g for 15 mins to collect the upper supernatant. Plasma samples were stored at -20°C and avoid repeated freeze-thaw cycles. The fluorescent coded microspheres (RK05203, ABclonal Technology) were combined and mixed with supernatants following the manufacturer’s instructions with the assistance of ABclonal Technology. Two technical replicates were designed per plasma sample.

### 2.14 RNA sequence

MACS-CD19^+^ cells were isolated from the bone marrow of wild type C57BL/6 mice and treated with PBS (Control group) or tEVs (Experimental group) for 24 hours. RNA sequencing was performed on samples from both groups using Illumina’s next-generation sequencing platform. Differentially expressed genes (DEGs) (**Supplementary Table S3**) were identified as statistically significant using thresholds of P-values ≤ 0.05 and |log2FC| ≥1.

### 2.15 Statistical analysis

All data were analyzed using Prism 8 (GraphPad Software). Results are expressed as mean ± standard error of the mean (SEM). Comparisons between two groups were performed using unpaired Student’s t-tests. One-way ANOVA was used to analyze three or more groups for statistical significance. The correlation between mTOR and RAB1A was examined using Spearman’s correlation analysis. P < 0.05 was considered statistically significant (*, P < 0.05; **, P < 0.01; ***, P <0.001) and ns indicates non-significant differences.

## 3 Results

### 3.1 Inactivation of the mTOR signaling pathway accelerates RAB1A degradation via the ubiquitin-proteasome system

**Figure 1:**
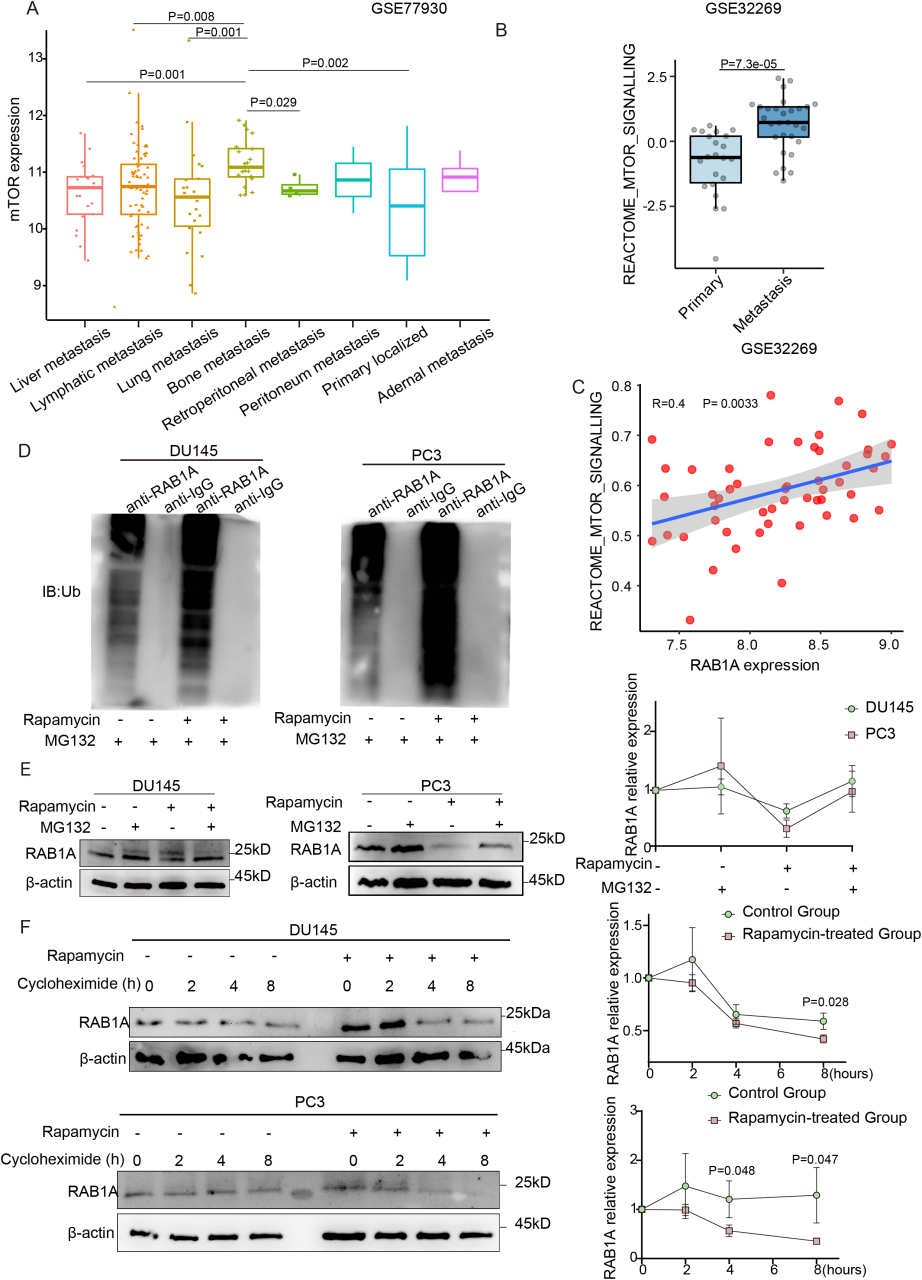
Activation of mTOR pathway promotes RAB1A protein stabilization via the decrease of ubiquitination. **(A)** Expression of mTOR in PCa patients with various metastatic sites (GSE77930 dataset). **(B)** Comparative analysis of mTOR signaling activity between localized PCa patients and bone metastasis cases (GSE32269 dataset). **(C)** Correlation between the mTOR signaling pathway and RAB1A expression in PCa patients from GSE32269. **(D)** PCa cells were treated with rapamycin (100 nM) or PBS for 8 hours and then subjected to Co-IP with an anti-RAB1A and immunoblot with Abs against Ub. **(E)** WB and quantification of RAB1A expression in PCa cells treated with or without MG132 (10 mM) for 8 hours. **(F)** WB and quantification of RAB1A expression, with and without treatment with rapamycin (100 nM) and/or CHX (10 mM), in DU145 cells (top) and PC3 cells (bottom) harvested at the indicated time points. **(E, F)** The experiments were repeated independently at least three times. Data were analyzed using t-test and one-way ANOVA with multiple comparisons test **(A)**. The correlation between mTOR and RAB1A was examined using Spearman’s correlation analysis **(C)**. P < 0.05 was considered statistically significant.

We analyzed mTOR mRNA expression across metastatic CRPC subtypes using the GSE77930 dataset. Bone metastatic tumor tissues exhibited significantly elevated mTOR expression levels compared to other sites (**Figure 1A**). To further clarify the association between mTOR signaling pathway activity and bone metastasis, we revealed that tumor tissues from PCa patients with bone metastasis exhibited significantly elevated mTOR signaling pathway activity compared to localized-stage PCa using the GSE32269 dataset (**Figure 1B**). Notably, there was also a modest positive association between pathway activity and RAB1A expression (R = 0.4, P = 0.0033; **Figure 1C**). Given previous reports of RAB1A-mediated mTOR regulation in colorectal cancer (24), we downregulated RAB1A expression of two human PCa cell lines with RAB1A-targeting shRNA lentiviruses (**Supplementary Figure S1A**). The results revealed that RAB1A knockdown did not alter S6K phosphorylation status, contrasting findings from Thomas’s study (**Supplementary Figure S1B**). However, mTOR inhibition via rapamycin treatment reduced RAB1A protein levels in PCa cells, while serum starvation (FBS removal) showed no effect on RAB1A expression (**Supplementary Figure S1C**). Next, consistent with public datasets, we confirmed elevated RAB1A expression in PC3 cells (derived from bone metastasis) compared to DU145 cells (originating from brain metastasis) (**Supplementary Figure S1D**).

Our previous study reported RAB1A protein modification in PCa cells (17). Thus, we hypothesized that the mTOR signaling pathway regulated RAB1A at the protein level via the ubiquitin-proteasome system. Co-IP assays showed that mTOR indeed physically interacted with RAB1A (**Supplementary Figures S1E, F**). Co-treatment of PCa cell lines with rapamycin and the proteasome inhibitor MG132 restored RAB1A protein levels that were reduced by rapamycin alone. Notably, the mTOR inhibitor induced a further prominent increase in RAB1A ubiquitination (**Figure 1D-E**). To investigate RAB1A stability under rapamycin treatment, we performed half-life analyses using the protein synthesis inhibitor CHX. These experiments revealed that RAB1A protein was significantly more stable in the absence of rapamycin treatment *in vitro* (**Figure 1F**). Mechanistically, the mTOR signaling pathway was shown to regulate RAB1A post-translational modification in a ubiquitin-dependent manner.

### 3.2 RAB1A expression is positively correlated with PCa progression

**Figure 2:**
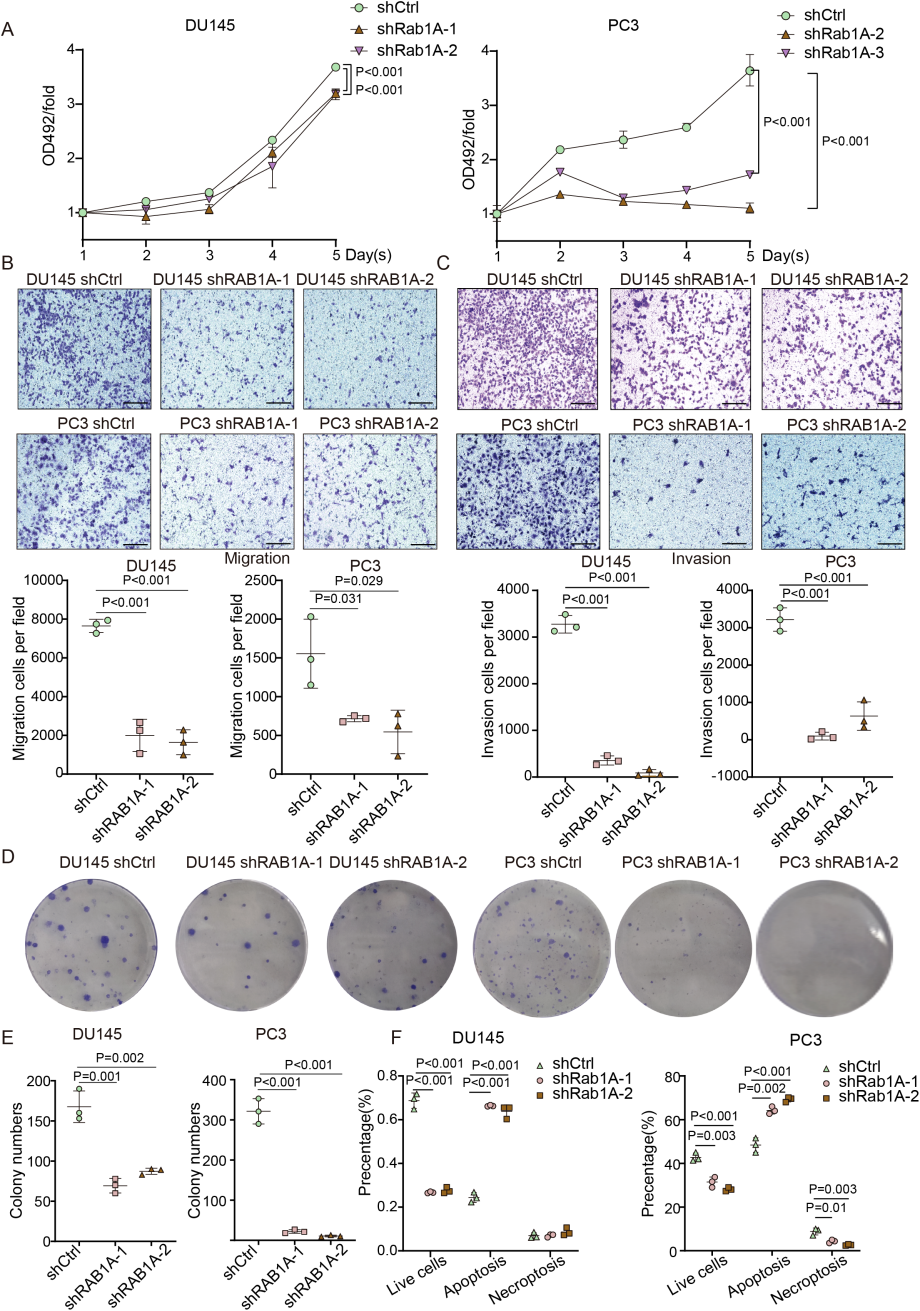
RAB1A overexpression is essential for PCa growth. **(A)** Growth of human PCa cell lines following RAB1A knockdown, as quantified by MTS assay. **(B)** The effect of RAB1A expression on PCa cell migration according to the transwell assay in PCa cell lines (scale bar: 200µm). **(C)** Invasion assay of PCa cell lines transfected with shCtrl or shRAB1A lentivirus in PCa cells (scare bar: 200µm). **(D)** Colony formation in shCtrl and RAB1A stably knockdown PCa cells plated at an initial density of 1,000-2,000 cells. **(E)** Statistical analysis of colony formation assays. **(F)** Evaluation of apoptotic cells in PCa cell lines transfected with shCtrl or shRAB1A lentivirus. Data are presented as means ± SD (n=3). Differences between the groups were analyzed by one-way ANOVA. P < 0.05 was considered statistically significant.

We further investigated the impact of RAB1A on PCa by reducing its expression. Next, RAB1A knockdown significantly impaired the proliferation of both PCa cell lines (**Figure 2A**). Consistently, silencing of RAB1A led to significant reductions in the migration and invasion capacity of PCa cells (**Figures 2B, C**), and inhibited colony formation, as shown by colony formation assays (**Figures 2D, E**). Additionally, depleting RAB1A resulted in higher proportions of apoptotic cells using propidium iodide (PI)/annexin V staining (**Figure 2F**). Overall, our findings suggested that RAB1A knockdown attenuated tumor development, indicating that RAB1A might have potential as a therapeutic target to inhibit PCa development.

### 3.3 RAB1A expression positively influences EV production

**Figure 3:**
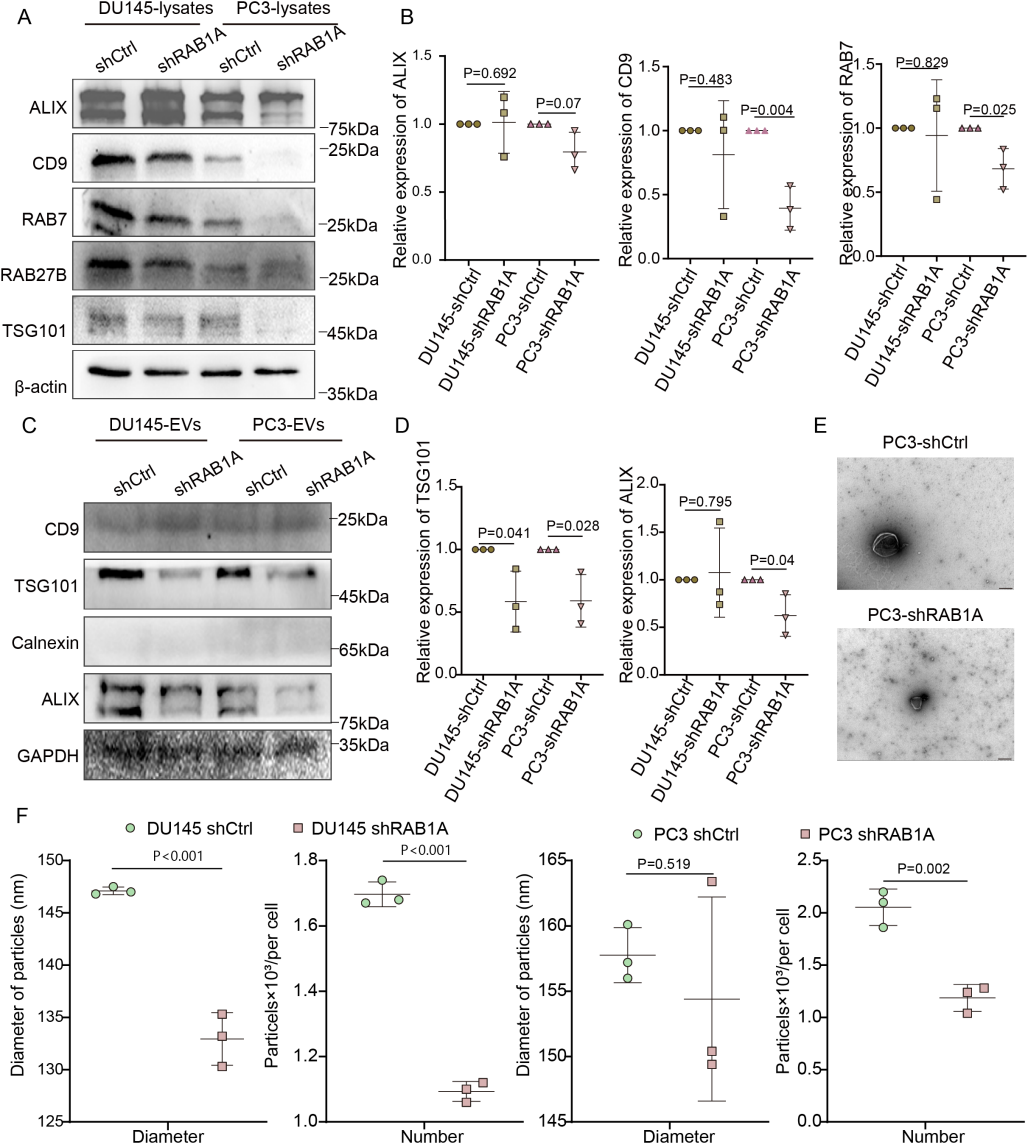
RAB1A knockdown influences EV secretion in PCa. **(A)** Expression of EV-associated genes was evaluated in PCa cells. **(B)** Quantitative analysis of ALIX, CD9, and RAB7 protein expression in shCtrl and shRAB1A PCa cells. **(C)** Expression of EV-related genes in EVs derived from shCtrl and shRAB1A PCa cells. **(D)** Quantification of ALIX, CD9, and RAB7 protein expression in EVs derived from shCtrl and shRAB1A PCa cells. **(E)** Representative TME images of EVs isolated from shCtrl and shRAB1A PC3 cells (scale bar: 100 nm). **(F)** Size distribution analysis of EVs derived from shCtrl and shRAB1A PCa cells by NTA. All western blot images have been repeated at least three times. Data were analyzed using t-test. P < 0.05 was considered statistically significant.

To elucidate the potential downstream regulatory mechanisms, we analyzed differentially expressed genes (DEGs) between high- and low RAB1A expressing groups based on the median RAB1A mRNA using TCGA database data. The results showed that RAB1A was associated with a variety of genes involved in small GTPase-mediated signal transduction (**Supplementary Figure S2A**). It is well acknowledged that the Rab GTPase family is crucial in vesicular trafficking and EV secretion, with secretory vesicles linked to actin cytoskeletal dynamics (7). To investigate the impact of RAB1A knockdown on EV biogenesis, we screened a panel of EV-associated factors (7, 25, 26). As shown in **Supplementary Figure S2B**, qRT-PCR analysis revealed significant downregulation of mRNA expression for selected factors (such as RAB27, RAB7, ALIX, RAB5, RAB11, SNAP23, RAB35). We further profiled selected factors (CD9, RAB7, ALIX, RAB27, TSG101) and validated their expression at the protein level (**Figure 3A**). WB analysis demonstrated reduced protein expression to varying degrees in shRAB1A PCa cells (**Figure 3B**, **Supplementary Figure S2C**). Consequently, these data establish RAB1A as a key regulator of EV biogenesis in PCa, likely acting through the coordinated suppression of a network of essential Rab GTPases (RAB27A, RAB7A, RAB5A, RAB11A, RAB35) and EV cargo-sorting/scaffolding factors (ALIX, TSG101, SNAP23, CD9).

We further validated that the typical markers for tEVs and EVs derived from shRAB1A PCa cells expressed lower levels of TSG101 and Alix at the same concentration (**Figure 3C**, **Supplementary Figure S2D**). TEM images showed invaginations and typical cup-shaped membrane vesicles (**Figure 3D**, **Supplementary Figures S3A, E**). Nanoparticle tracking analysis (NTA) showed a particle distribution within the 100–200 nm range, consistent with the diameter of functional EVs. shRAB1A PCa cells secreted fewer EVs with a smaller size compared to control cells (**Figure 3E**, **Supplementary Figures S3C, D**). These findings collectively demonstrate that RAB1A knockdown significantly disrupts EV biogenesis and secretion in PCa cells. The concomitant decrease in both EV yield and particle size further indicates the essential role of RAB1A in regulating EV quantity and size characteristics.

### 3.4 B lymphocyte-myeloid cell ratio imbalance in the pre-metastatic bone microenvironment

**Figure 4:**
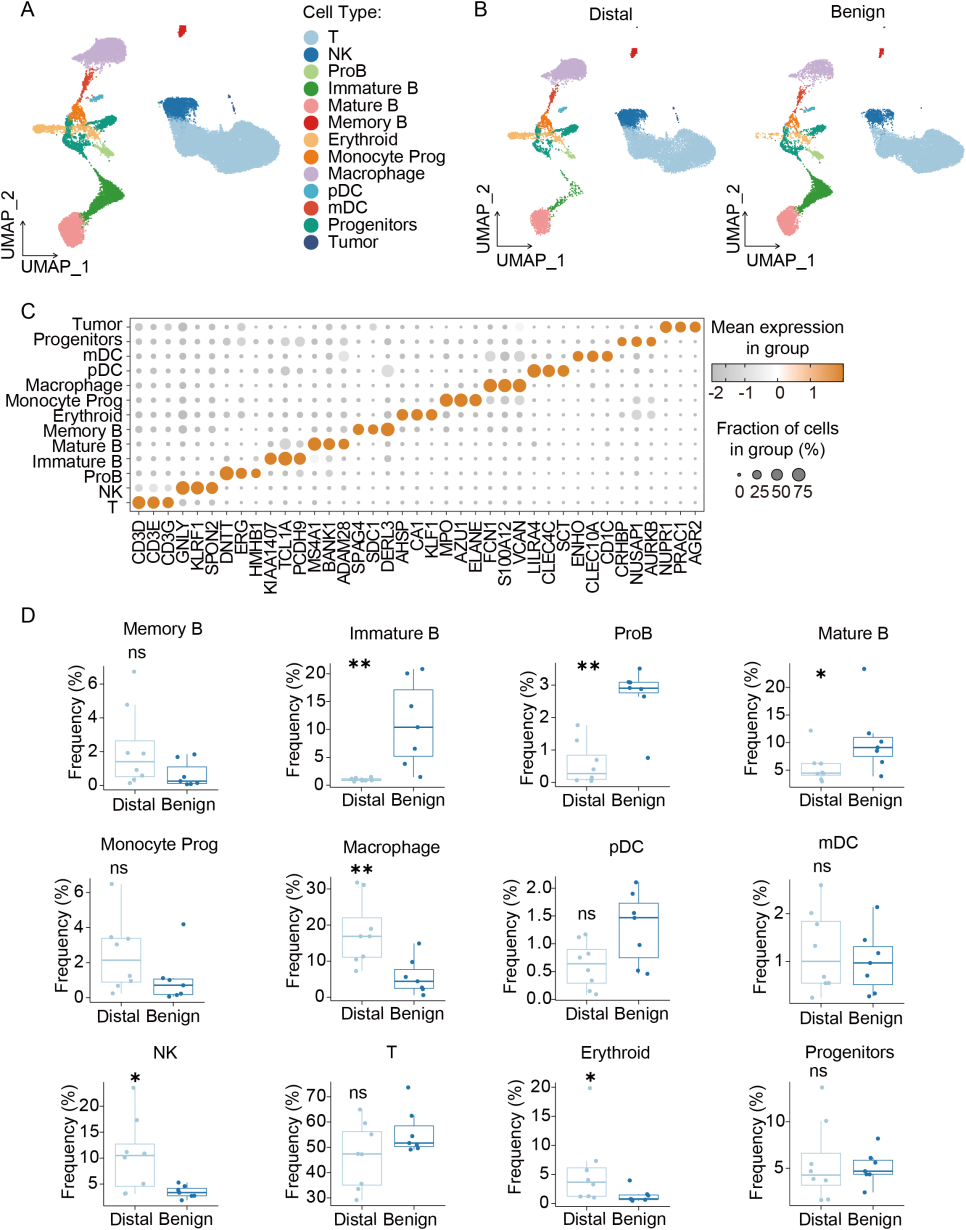
Overview of compositional shifts in Distal Group associated with bone metastases in PCa patients. **(A)** Comparative analysis of bone marrow cellular composition between Distal samples from metastatic PCa patients (n=8) and Benign controls (n=7) using integrated scRNA-seq data. **(B)** Compositional alterations in bone marrow cells between the two groups were visualized on a UMAP embedding. **(C)** Marker genes for major cell populations. **(D)** Quantitative comparison of principal bone marrow cell population frequencies between groups. All experiments have been repeated at least three times. Data were analyzed using t-test. P < 0.05 as *; P < 0.01 as ** and ns as not significant.

Primary tumors remotely reprogrammed an immunosuppressive microenvironment in the bone marrow niche by secreting many soluble molecules, providing the subsequent CTCs with a notable survival advantage (10, 27). Based on the GSE143791 single-cell dataset, we analyzed the sequencing data of bone marrow cells from patients undergoing hip replacement surgery (*Benign Group*) and different vertebral specimens distant from the tumor site (*Distal Group*). As shown in **Figure 4A**, quality control and normalization were performed on the two groups. The bone marrow cell subpopulation atlas revealed decreased B cell populations and increased macrophages (**Figure 4B**). The statistical results shown in **Figure 4C** indicated that the proportion of T cells in *Distal Group* showed no significant difference compared to the *Benign Group*, while the proportions of progenitor B cells, immature B cells, and mature B cells were all significantly reduced (P < 0.05). In contrast, the proportions of macrophages and NK cells were significantly increased (P < 0.05). Furthermore, the proportion of erythroid in the *Distal Group* was significantly increased (P < 0.05). In conclusion, prior to the formation of bone metastatic lesions, the bone marrow microenvironment had already participated in the PMN construction through various regulatory mechanisms, characterized by myeloid cell expansion and suppressed development of the B-lymphocyte lineage.

### 3.5 EVs derived from PCa dysregulate the proportions of lymphocytes and myeloid cells

**Figure 5:**
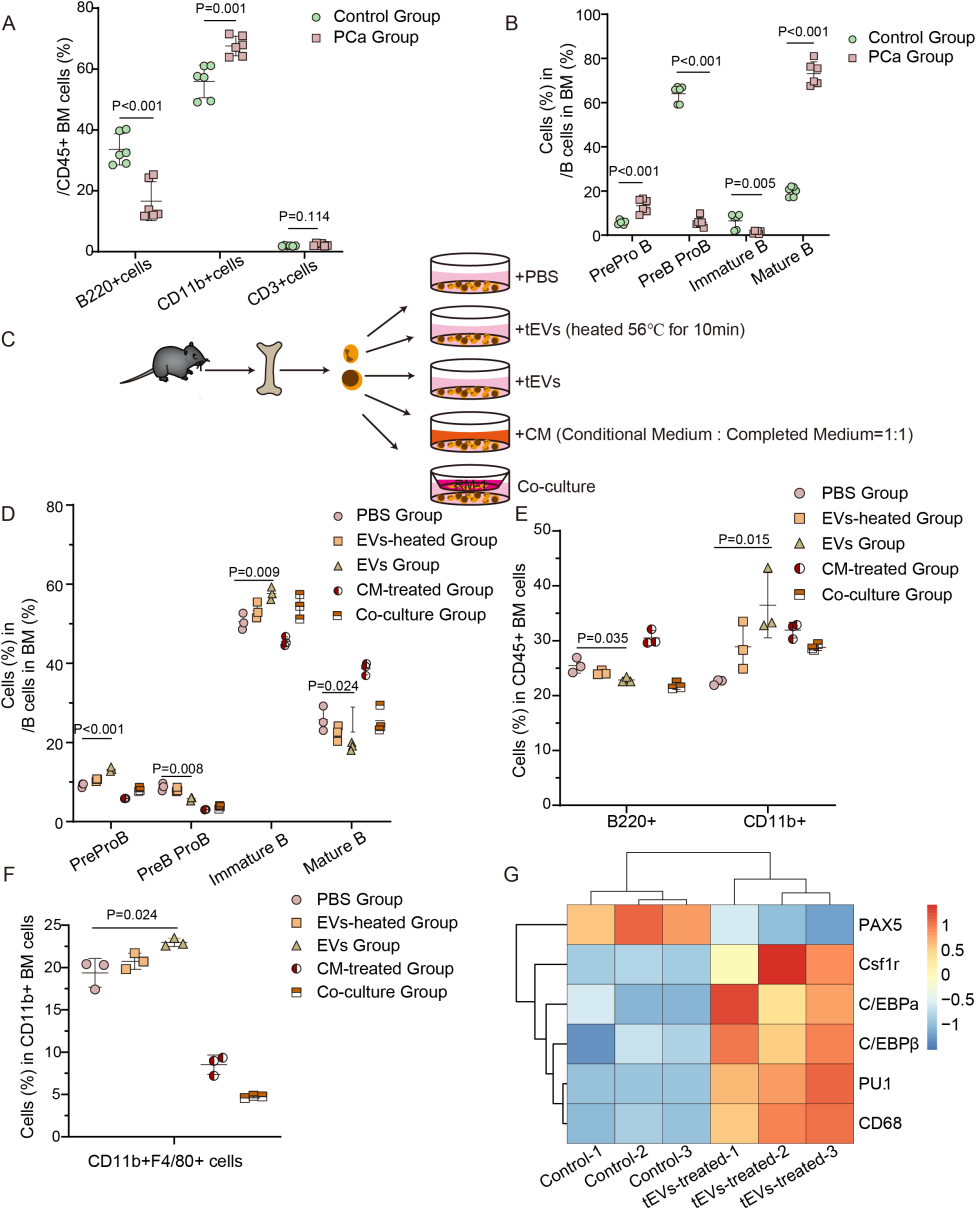
EVs derived from tumor induce B lymphocyte-myeloid cell imbalance in bone marrow. **(A)** Flow cytometry analysis of bone marrow cell subtypes in PCa and control groups. **(B)** Quantification of B cell subtype distribution in bone marrow in PCa and control groups. **(C)** Schematic of the *in vitro* co-culture model for tEV-bone marrow cell interactions. **(D)** Quantification of B cell subtype *in vitro* culture systems. **(E)** Frequencies of B220^+^CD11b^−^ (B lineage), and B220^−^CD11b^+^ (myeloid lineage) cells *in vitro* culture systems. **(F)** Frequency of CD11b+F4/80^+^ macrophages within adherent cells. **(G)** qRT-PCR screen for representative TFs indicated in progenitor cell differentiation in the presence or absence of tEVs. All experiments have been repeated at least three times. Data were analyzed using t-test **(A, B)** and one-way ANOVA with multiple comparisons test **(D-F)**. P < 0.05 was considered statistically significant.

Compared to orthotopic models with low spontaneous metastasis rates (22), LCV injection directly targets skeletal dissemination, enabling effective modelling of the PCa bone metastasis immune microenvironment. Therefore, we established a mouse model of bone metastasis via LCV injection of RM-1 cells (**Supplementary Figure S4A**). Analysis of bone marrow cell subtypes in PCa mice with bone metastasis revealed a reduction in total B cells, an increase in CD11b^+^ cells, and no significant change in CD3^+^ cells, indicating severe disruption of bone marrow cell development (**Figure 5A**). Flow cytometry further characterized all major B-cell subsets in the bone marrow (**Supplementary Figure S4B**). To investigate the regulatory role of tEVs in bone marrow cell differentiation, we treated bone marrow cells with tEVs, heated-inactivated tEVs (heated at 56°C for 10 min to effectively denature the bioactive molecules), conditioned medium, or co-cultured them with RM-1 cells (**Figure 5C**). EVs-treated bone marrow cells demonstrated an increase in the frequency of pre-pro B cells (B220^lo+^CD24^+^BP-1^-^), alongside a decrease in pro-B/pre-B cells (B220^+^CD24^+^CD43^-^IgM^-^IgD^-^) both *in vivo* (**Figure 5B**) and *in vitro* (**Figures 5C-E**). These alterations correlated positively with tEV concentrations (**Supplementary Figure S4C**) and tEVs block the differentiation of pre-pro-B cells into pro-B/pre-B cells, which was attenuated by heat inactivation. Considering the aforementioned finding that RAB1A knockdown decreased EV production, we treated bone marrow cells with EVs derived from the equal number of RM-1 cells transduced with either control shRNA (shCtrl-EVs) or RAB1A-targeting shRNA (shRAB1A-EVs). Consistent with the concentration-dependence, shRAB1A-EVs (produced in lower quantities) induced less pronounced B-cell population shifts compared to shCtrl-EVs (**Supplementary Figure S4D**).

*In vitro* studies (**Figure 5D**) showed a significant decrease in B lymphocytes and an increase in CD11b^+^ myeloid cells, which was consistent with our *in vivo* observation (**Figure 5A**). Furthermore, there was a significant number of adherent cells after tEV treatment, most of which were CD11b^+^F4/80^+^ cells (**Figure 5F**, **Supplementary Figure S4E**). Analysis of hematopoietic transcription factors (TFs) (28, 29) revealed that tEVs reprogrammed the transcriptional profile of bone marrow cells towards a myeloid lineage, evidenced by decreased expression of PAX5 (a B-cell master regulator) and increased expression of myeloid-associated genes (**Figure 5G**). Collectively, tEVs induce a myeloid-biased signature in bone marrow cells, driven by both increased CD11b^+^ cell abundance and TF-mediated transcriptional reprogramming.

### 3.6 B cells transdifferentiate into myeloid cells under tEV-mediated induction

**Figure 6:**
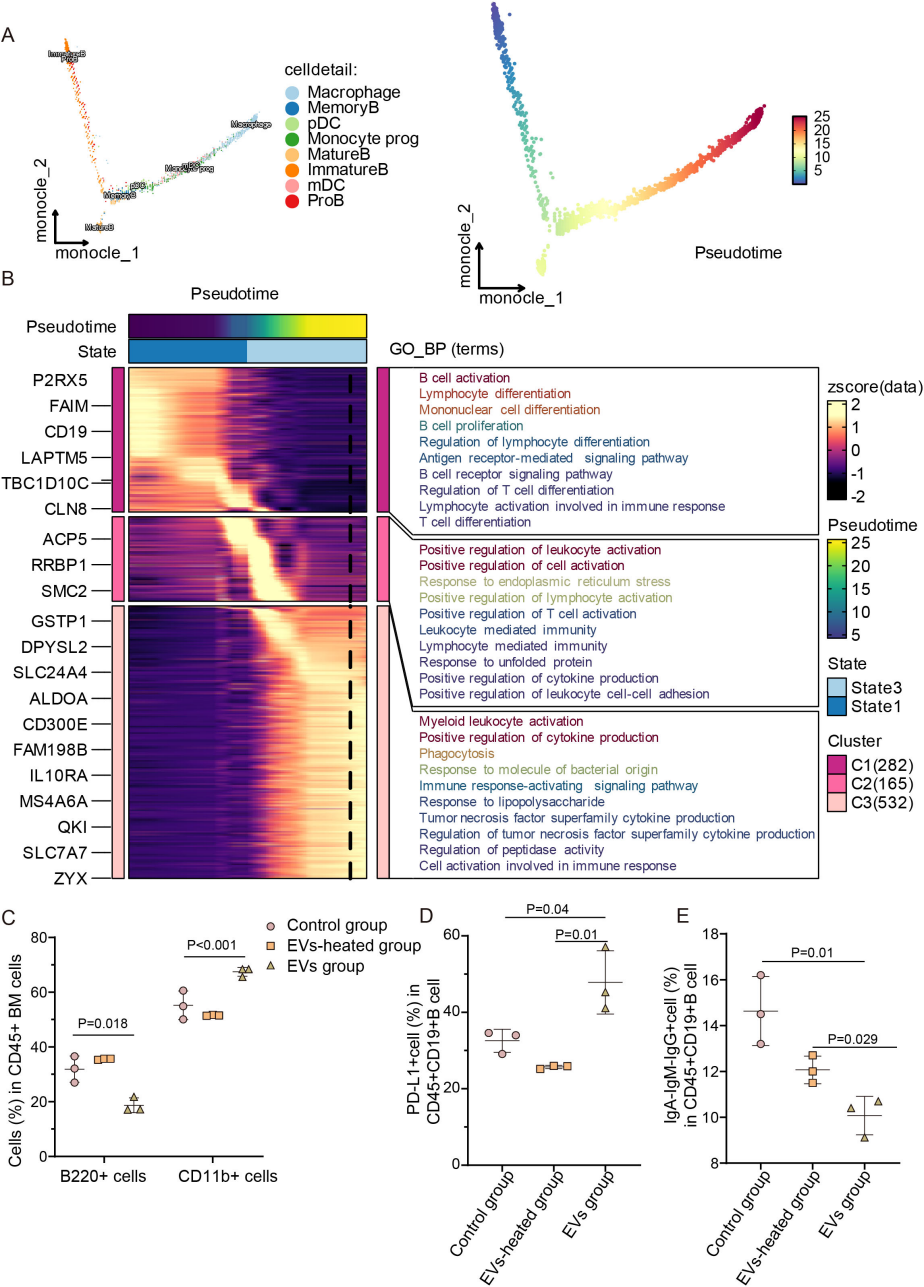
EVs derived from tumor induced CD19^+^ B cells transdifferentiation. **(A)** Unsupervised transcriptional trajectory of B lymphocytes and myeloid cells generated by Monocle2, colored by cell states and subsets. **(B)** Dynamic heatmap patterns revealing critical cellular developmental transitions. **(C)** Frequencies of B220^+^ B cells and CD11b^+^ myeloid cells in mouse bone marrow (n=3). **(D)** Frequency of PD-L1^+^ B cells among CD45^+^CD19^+^ B cells in bone marrow (n=3). **(E)** Proportion of IgA^−^IgM^−^IgG^+^ B cells among CD45^+^CD19^+^ B cells in bone marrow (n=3). **(C-E)** Experiments have been repeated at least three times. Data were analyzed using one-way ANOVA with multiple comparisons test **(C-E)**. P < 0.05 was considered statistically significant.

Multi-color IF staining for CD19 and CD68 revealed the presence of a small population of CD19^+^ cells within CD68^+^ myeloid cells in the bone metastatic sites of PCa models, suggesting a potential interaction between CD19^+^ B cells and CD68^+^ macrophages (**Supplementary Figure S5A**). Myeloid and lymphoid lineages arise from common multipotent progenitors and share a similar set of genes in their differentiation processes (30). Under certain circumstances, the response to the ever-changing needs of the hematopoietic system can lead to transformation between the two lineages (31). Single-cell trajectory analysis of sorted bone marrow cells demonstrated B-myeloid transdifferentiation within the bone PMN (**Figure 6A**). Subsequently, we employed pseudotemporal heatmap analysis to identify critical cell-state transition points and associated signaling pathways orchestrating stage-specific differentiation (**Figure 6B**).

To relatively mimic the condition for tEVs production at the PMN formation phase, we treated C57BL/6 mice with PBS, heat-inactivated tEVs, or tEVs through tail vein injection. First, we administered PKH26-labeled tEVs intravenously and analyzed bone marrow cells by flow cytometry after 4 hours (**Supplementary Figure S5B**). As shown in **Figure 6C**, bone marrow cells from tEVs-treated mice exhibited significant enrichment of myeloid cells and a sharp decrease in B lymphocytes. Furthermore, tEVs induced PD-L1^+^ Breg within bone marrow and significantly inhibited B cell activation (**Figures 6D, E**). Correspondingly, in the PB of the *tEVs-treated Group*, the total B cells were significantly decreased, while myeloid cells were increased, which mirrored the trends observed in the bone marrow (**Supplementary Figure S5C**). Simultaneously, the proportion of PD-L1^+^ Breg cells increased, and B cell activation status remained suppressed in bone marrow cells and PB cells (**Supplementary Figures S5D, E**). In addition, we found that the plasma cytokine profile in the tEVs group manifested pro-tumor characteristics, such as increased IL-6 and decreased IL-12p40, IL-12p70 (**Supplementary Figure S5F**) (32). Treatment with tEVs led to slower growth in body weight, although the difference did not reach statistical significance (**Supplementary Figure S5G**). Systemic administration of active tEVs (but not heat-inactivated tEVs) in mice recapitulated PMN formation.

MACS-purified CD19^+^ B cells (> 95% purity; **Supplementary Figure S6A**) were cultured in a complete medium supplemented with tEVs. EV exposure induced significant morphological alterations in CD19^+^ B cells, manifested as increased cell size (quantified by FSC-A) (**Supplementary Figure S6B**). As shown *in vivo* (**Figure 6C**) and *in vitro* (**Supplementary Figure S6C**), B220^+^CD11b^-^ cells were increased in both proportion and absolute cell numbers in tEVs-treated groups. Next, we performed transcriptome sequencing on CD19^+^ B cells treated with PBS or tEVs. In tEVs-treated CD19^+^ cells, we observed increased expression of genes related to M2 macrophages (Arginase-1, Myc, and CD163) and chemokines (**Supplementary Figure S6D**). GO analysis showed that tEVs inhibited the activity of immunoglobulin production, B cell activation, adaptive immune response, immune cell proliferation, among other processes (**Supplementary Figure S6E**). Taken together, these findings demonstrated that tEVs drove B-cell transdifferentiation into myeloid lineages, which contributed to the imbalance between B lymphocytes and myeloid cells in PMN formation.

### 3.7 PCa-derived EVs promote bone metastasis *in vivo*

**Figure 7:**
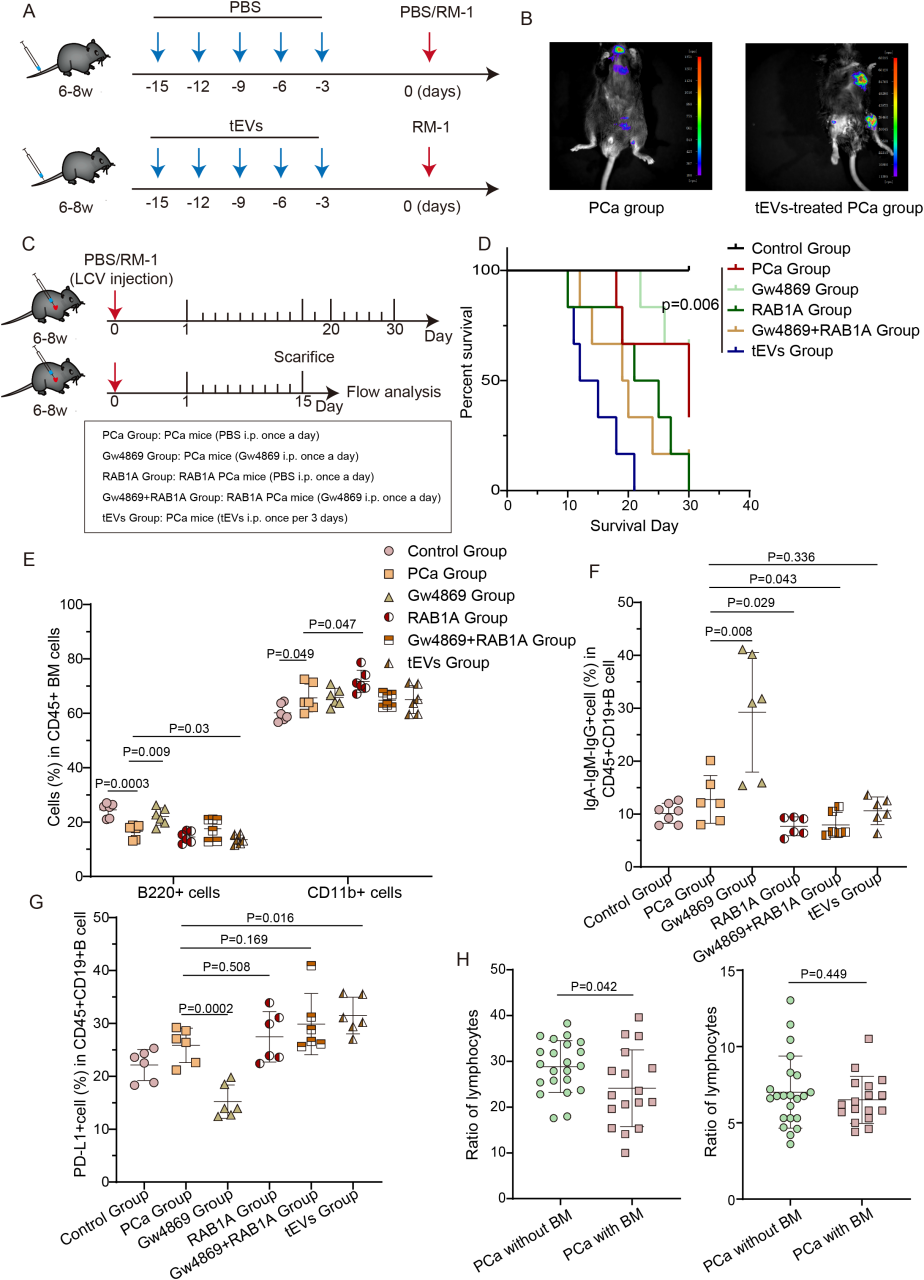
EVs-treated C57BL/6 mice exhibit reduced survival and accelerated bone metastasis. **(A)** Schematic of the experimental approach for establishing the PCa mouse model (n=6). **(B)** Bone metastatic load measured by luciferin photon flux. **(C)** Schematic of therapeutic intervention phase depicting RAB1A and GW4869 (n=6). **(D)** Survival time of PCa mouse models. **(E)** Frequencies of B220^+^ B cells and CD11b^+^ myeloid cells in mouse bone marrow. **(F)** Frequency of PD-L1^+^ B cells among CD45^+^CD19^+^ B cells in bone marrow. **(G)** Proportion of IgA^−^IgM^−^IgG^+^ B cells among CD45^+^CD19^+^ B cells in bone marrow. **(H)** Quantification of lymphocytes and monocytes in bone marrow of treatment-naive PCa patients with (n=17) or without bone metastasis (n=22). BM, bone metastasis. Data were analyzed using t-test **(E-G)** and one-way ANOVA with multiple comparisons test **(H)**. P < 0.05 was considered statistically significant.

Next, we investigated the impact of tEV treatment on survival outcomes in a PCa model mice (**Figure 7A**). Given that intracardiac PCa models inadequately recapitulate early metastatic events that occur in the primary tumor, we pretreated wild type mice with tEVs (22). As expected, tEVs-pretreated PCa mice exhibited reduced survival and increased bone metastatic burden (**Supplementary Figure S7A**, **Figure 7B**). Quantitative analysis revealed a significant increase in CD11b^+^ myeloid cell frequencies accompanied by a concomitant decrease in B lymphocyte populations within the bone marrow of tEVs-treated PCa mice (**Supplementary Figure S7B**). Furthermore, tumor-bearing mice exhibited splenomegaly whereas tumor-free control mice did not (not shown). To elucidate the potential contribution of RAB1A to the immune modulation of cancer immunoediting, we used RAB1A overexpression plasmid and GW4869, an inhibitor of exosome biogenesis to establish the experimental model (33). As shown in **Figure 7E**, GW4869 significantly restored the RAB1A-induced imbalance between myeloid cell and B cell. Simultaneously, we observed increased PD-L1^+^ Breg proportion (**Figure 7G**) and suppression of B-cell activation in bone marrow (**Figure 7F**). These results demonstrated that RAB1A promotes PCa progression by enhancing EV secretion, whereas inhibition of EV secretion restores the bone marrow microenvironment and suppresses tumor progression. Clinically, PB analysis revealed significantly reduced lymphocyte proportions (P = 0.042) but unchanged monocyte frequencies in PCa patients with bone metastasis compared to those without metastasis (**Figure 7H**). Collectively, these findings establish the RAB1A-EV secretion axis as a master regulator of bone metastatic niche formation, driving immunosuppression via myeloid expansion and B-cell dysfunction, while its therapeutic blockade offers a multifaceted strategy to disrupt the vicious cycle of PCa progression.

## 4 Discussion

At the early stage of bone metastasis development, tEVs are released into the blood circulation and transported to the bone, where they orchestrate an immunosuppressive and inflammatory PMN microenvironment through multifaceted molecular mechanisms (34, 35). Since that discovery, extensive research focused on characterizing the formation of the pre-metastatic microenvironment, primarily initiated by regulatory immune cells (21, 36). For instance, exosomes derived from melanoma-educated bone marrow progenitor cells promote a pro-metastatic phenotype (37). Notably, our prior work revealed a novel mechanism by which EVs derived from esophageal cancer disrupted the equilibrium between circulating follicular helper T and circulating follicular regulatory T cells, thereby promoting immune escape and tumor progression (20). Consequently, it was uncertain whether tEVs could indirectly modulate the function of other immune cells via B cells, which had remained a huge knowledge gap in understanding tEVs-B cell crosstalk during PMN formation (8).

In our study, we confirmed that mTOR expression was elevated in PCa patients with bone metastasis compared to those with other metastases. Here, we found that RAB1A expression decreased in response to inactivation of the mTOR signaling pathway, which may elucidate the limited clinical efficacy of mTOR inhibitors in PCa treatment. Additionally, activation of the mTOR signaling pathway upregulated RAB1A expression and thus promoted EV release. Mice injected with tEVs exhibited significant reductions in B-lymphoid populations alongside expanded myeloid compartments, mirroring the immune cell pattern found in the bone microenvironment of the vertebral body distant from the tumor site in human patients (9). These findings establish tEVs as direct mediators of bone marrow immunomodulation in metastatic progression.

In the bone marrow, B-cell precursors and immature IgM^+^ B cells retain plasticity and the potential for myeloid transdifferentiation (38–40). In addition, tEVs drive bone marrow reconstitution through myeloid differentiation and disruption of lymphoid-biased HSC development. Although our results demonstrated that tEVs could induce transdifferentiation of B cells into myeloid cells *in vivo* and *in vitro*, they also revealed variations in the heterogeneity and plasticity of cells at the early stage of bone metastasis. Nevertheless, the specific factors carried by the tEVs that account for the dysregulation of lymphopoiesis and myelopoiesis are unclear. It is reported that the enrichment of TGF-β and miR-21 in EVs provides candidate mediators for functional validation (41, 42). Further studies should employ EV-subtype fractionation and CRISPR-based cargo editing to establish causality. These findings establish tEVs as direct mediators of bone marrow immunomodulation in metastatic progression.

Collectively, our study delineates a novel mTOR/RAB1A-regulated EV secretion axis that orchestrates PCa bone metastasis. EVs drive immunosuppressive niche formation via B-lymphocyte suppression and myeloid skewing. The results of our *in vivo* experiments emphasized the significant role of tEVs in bone metastasis progression in PCa. In addition, RAB1A as a novel target with the potential to reverse immunosuppression and enhance immunotherapeutic responses in clinical practice. The limited efficacy of mTOR inhibitors in PCa may reflect compensatory EV-mediated crosstalk, suggesting that co-targeting RAB1A and PD-1/PD-L1 could overcome microenvironmental immunosuppression. Considering that primary tumors disrupt bone marrow cell activities by releasing factors, cell subpopulations in PB may serve as potential biomarkers for detecting bone metastasis and monitoring responses to antiresorptive therapies. We will further include prospective correlation analyses of bone marrow-peripheral B-cell dynamics to quantify proportional shifts and subtype alterations alongside clinical evidences of bone metastasis in longitudinal PCa cohorts.

## Data Availability

The data presented in the study are deposited in the SRA database, accession number PRJNA1321719.

## Supplementary material

Supplementary Figure 1 Correlation between mTOR and RAB1A in PCa. **(A)** Analysis of PCa cells infected with lentiviral RAB1A shRNAs. **(B)** Analysis of p-S6K1 (T389) and total S6K in PCa cells infected with lentiviral RAB1A shRNAs. **(C)** Detection of RAB1A protein levels in PCa cell lines treated with rapamycin (100 nM) or serum-free culture medium for 24 h. **(D)** WB analysis of RAB1A expression in PCa cells. **(E, F)** Co-IP analysis of mTOR and RAB1A in PCa cells. Normal IgG was used as a negative control. All experiments have been repeated at least twice. Data were analyzed using t-test **(D)** and one-way ANOVA with multiple comparisons test. P < 0.05 was considered statistically significant.

Supplementary Figure 2 RAB1A knockdown affects EVs secretion in human PCa cell lines. **(A)** GO enrichment analysis of patients with high or low RAB1A expression from the TCGA prostate cancer dataset. **(B)** Expression levels of EV-related genes determined by PCR in shCtrl and shRAB1A PCa cells. **(C)** Protein expression of RAB27B and TSG101 in shCtrl and shRAB1A PCa cells. **(D)** CD9 protein expression levels in EVs derived from shCtrl and shRAB1A PCa cells. All experiments have been repeated at least three times. Data were analyzed using t-test **(B)** and one-way ANOVA with multiple comparisons test **(C, D)**. P < 0.05 was considered statistically significant.

Supplementary Figure 3 RAB1A affects EV secretion in RM-1 cells. **(A)** Knockdown efficiency of RAB1A in RM-1 cells. **(B)** Statistical analysis of RAB1A knockdown efficiency. **(C)** NTA results of EVs secreted by shCtrl and shRAB1A RM-1 cells. **(D)** Statistical analysis of NTA. **(E)** Representative TEM images of EVs (scale bar: 100 nm). All experiments have been repeated at least three times. Data were analyzed using t-test. P < 0.05 was considered statistically significant.

Supplementary Figure 4 Both tEVs and murine PCa cells dysregulate the proportions of lymphocytes and myeloid cells. **(A)** Representative H&E staining of bone from C57BL/6 mice with bone metastasis (scale bar: 50 µm). **(B)** Representative flow cytometric analysis of B subtypes in bone marrow. **(C)** Dose-dependent effects of tEVs on B cell subpopulations in cultured bone marrow cells (1×, 5 × 10^8^ particles/well; 2×, 1 × 10^9^ particles/well). **(D)** Quantified percentages of B cell subtypes in bone marrow cells cultured with shCtrl-EVs or shRAB1A-EVs. **(E)** Representative imaging of bone marrow cells after tEV treatment (scale bar: 20 µm). All experiments have been repeated at least three times. Data were analyzed using one-way ANOVA with multiple comparisons test **(C, D)**. P < 0.05 was considered statistically significant.

Supplementary Figure 5 EVs derived from PCa influence immune microenvironment. **(A)** IF imaging of tumor sections stained for CD68 (red), CD19 (green), and DAPI (blue) in PCa model with bone metastasis (scale bar: 200 µm). **(B)** Analysis of bone marrow cells by flow cytometry 4 hours after intravenous injection of PKH26-labeled tEVs. **(C)** Frequencies of B220^+^ B cells and CD11b^+^ myeloid cells in PB of mice (n=3). **(D)** Frequency of PD-L1^+^ B cells among CD45^+^CD19^+^ B cells in PB (n=3). **(E)** Proportion of IgA^−^IgM^−^IgG^+^ B cells among CD45^+^CD19^+^ B cells in PB (n=3). **(F)** ELISA assay performed on mouse plasma samples (n=3). **(G)** Body weight of mice. All experiments have been repeated at least three times. Data were analyzed using t-test and one-way ANOVA with multiple comparisons test **(A)**. P < 0.05 was considered statistically significant.

Supplementary Figure 6 EVs impair the development of MACS-CD19^+^ B cells *in vitro*. **(A)** Purity of MACS-purified CD19^+^ B cells. **(B)** Median FSC−A of CD19^+^ cell population in different groups. **(C)** Total count of myeloid cells (B220^-^CD11b^+^) calculated by flow cytometry in 1×10^6^ CD19^+^ cells treated with tEVs. **(D)** Volcano plot of DEGs. **(E)** GO enrichment analysis of DEGs downregulated in CD19^+^ cells treated with tEVs compared to control cells. All experiments have been repeated at least three times. Data were analyzed using one-way ANOVA with multiple comparisons test **(B, C)**. P < 0.05 was considered statistically significant.

Supplementary Figure 7 Pretreatment with tEVs shortens survival time and disrupts cell subtypes in mice. **(A)** Kaplan-Meier survival analysis of experimental mice. **(B)** Gating strategy for B cells and myeloid cells in bone marrow (top) and peripheral blood (bottom). **(C)** Quantification of B cells and myeloid cells in BM (left) and PB (right). Control Group, n=6; PCa Group, n=6; tEVs-treated PCa Group, n=4. All experiments have been repeated at least three times. Data were analyzed using one-way ANOVA with multiple comparisons test **(B)**. P < 0.05 was considered statistically significant.

Supplementary Table 1 Primers for mRNA expression analysis.

Supplementary Table 2 Flow antibodies.

Supplementary Table 3 DEGs between experimental groups and control groups.
