# Supplementary material for: Extracellular vesicles from prostate tumors reshape the pre-metastatic bone environment in an mTOR/RAB1A-dependent manner
Source: Front Immunol. 2025 Sep 19;16:1605494. doi: 10.3389/fimmu.2025.1605494 (PMC12491827; doi:10.3389/fimmu.2025.1605494)
Supplement: Supplementary Figure 1 — Correlation between mTOR and RAB1A in PCa. (A) Analysis of PCa cells infected with lentiviral RAB1A shRNAs. (B) Analysis of p-S6K1 (T389) and total S6K in PCa cells infected with lentiviral RAB1A shRNAs. (C) Detection of RAB1A protein levels in PCa cell lines treated with rapamycin (100 nM) or serum-free culture medium for 24 h. (D) WB analysis of RAB1A expression in PCa cells. (E, F) Co-IP analysis of mTOR and RAB1A in PCa cells. Normal IgG was used as a negative control. All experiments have been repeated at least twice. Data were analyzed using t-test (D) and one-way ANOVA with multiple comparisons test. P < 0.05 was considered statistically significant. [file DataSheet1.docx]

Supplementary Material

## Supplementary Figures


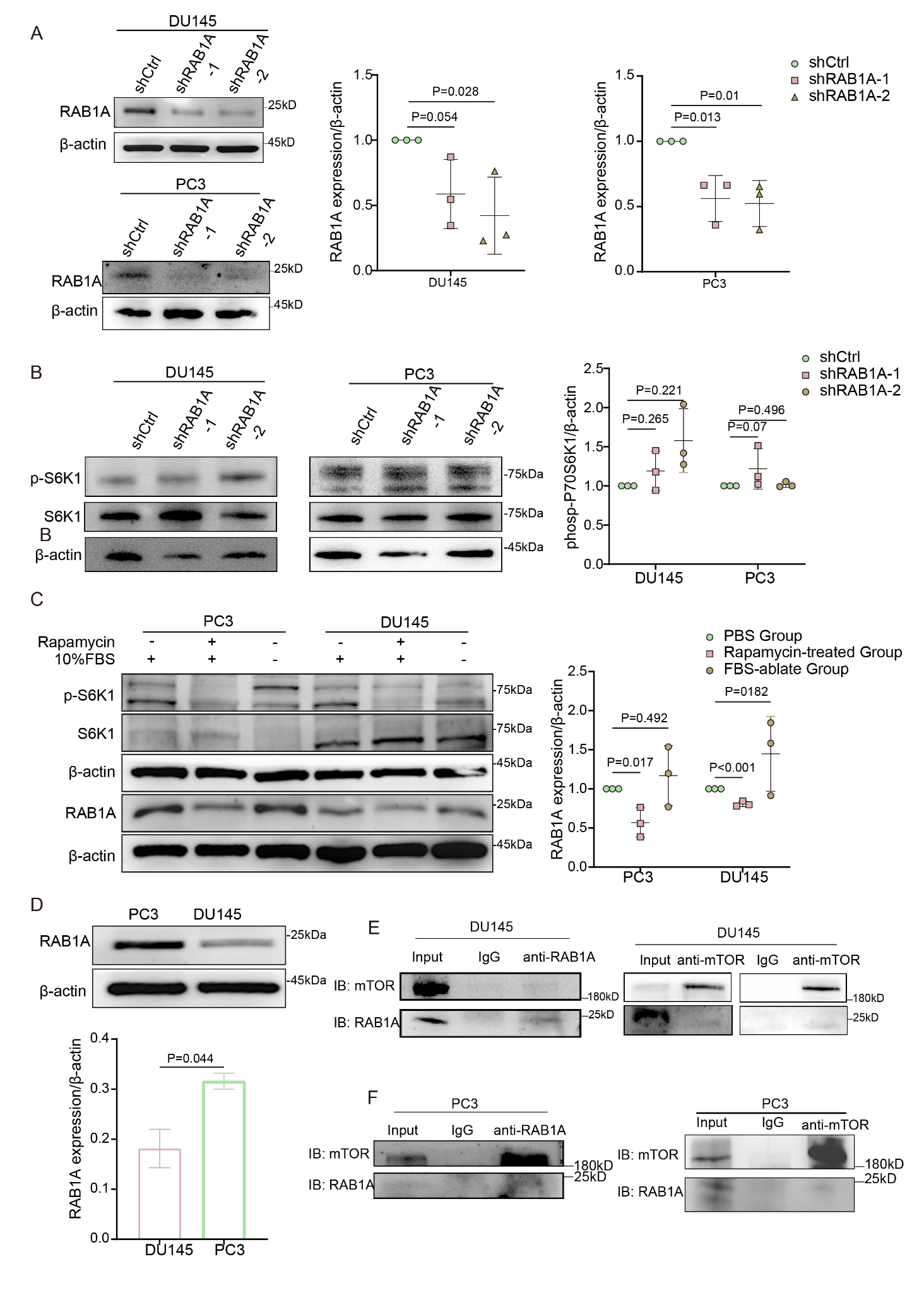


**Supplementary Figure 1.** Correlation between mTOR and RAB1A in PCa. (A) Analysis of PCa cells infected with lentiviral RAB1A shRNAs. (B) Analysis of p-S6K1 (T389) and total S6K in PCa cells infected with lentiviral RAB1A shRNAs. (C) Detection of RAB1A protein levels in PCa cell lines treated with rapamycin (100 nM) or serum-free culture medium for 24 h. (D) WB analysis of RAB1A expression in PCa cells. (E-F) Co-IP analysis of mTOR and RAB1A in PCa cells. Normal IgG was used as a negative control. All experiments have been repeated at least twice. Data were analyzed using t-test (D) and one-way ANOVA with multiple comparisons test. P < 0.05 was considered statistically significant.


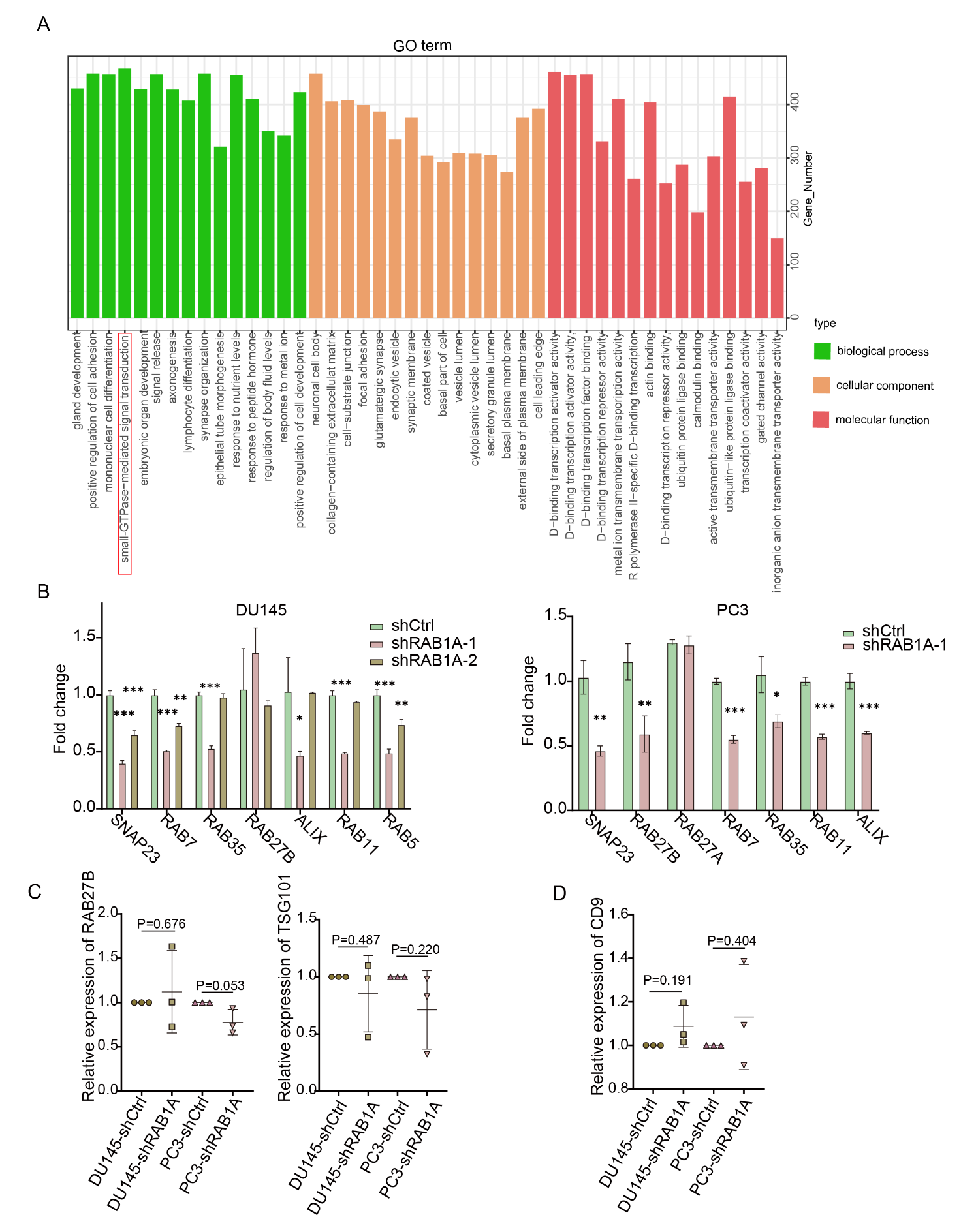


**Supplementary Figure 2.** RAB1A knockdown affects EVs secretion in human PCa cell lines. (A) GO enrichment analysis of patients with high or low RAB1A expression from the TCGA prostate cancer dataset. (B) Expression levels of EV-related genes determined by PCR in shCtrl and shRAB1A PCa cells. (C) Protein expression of RAB27B and TSG101 in shCtrl and shRAB1A PCa cells. (D) CD9 protein expression levels in EVs derived from shCtrl and shRAB1A PCa cells. All experiments have been repeated at least three times. Data were analyzed using t-test (B) and one-way ANOVA with multiple comparisons test (C-D). P < 0.05 was considered statistically significant.


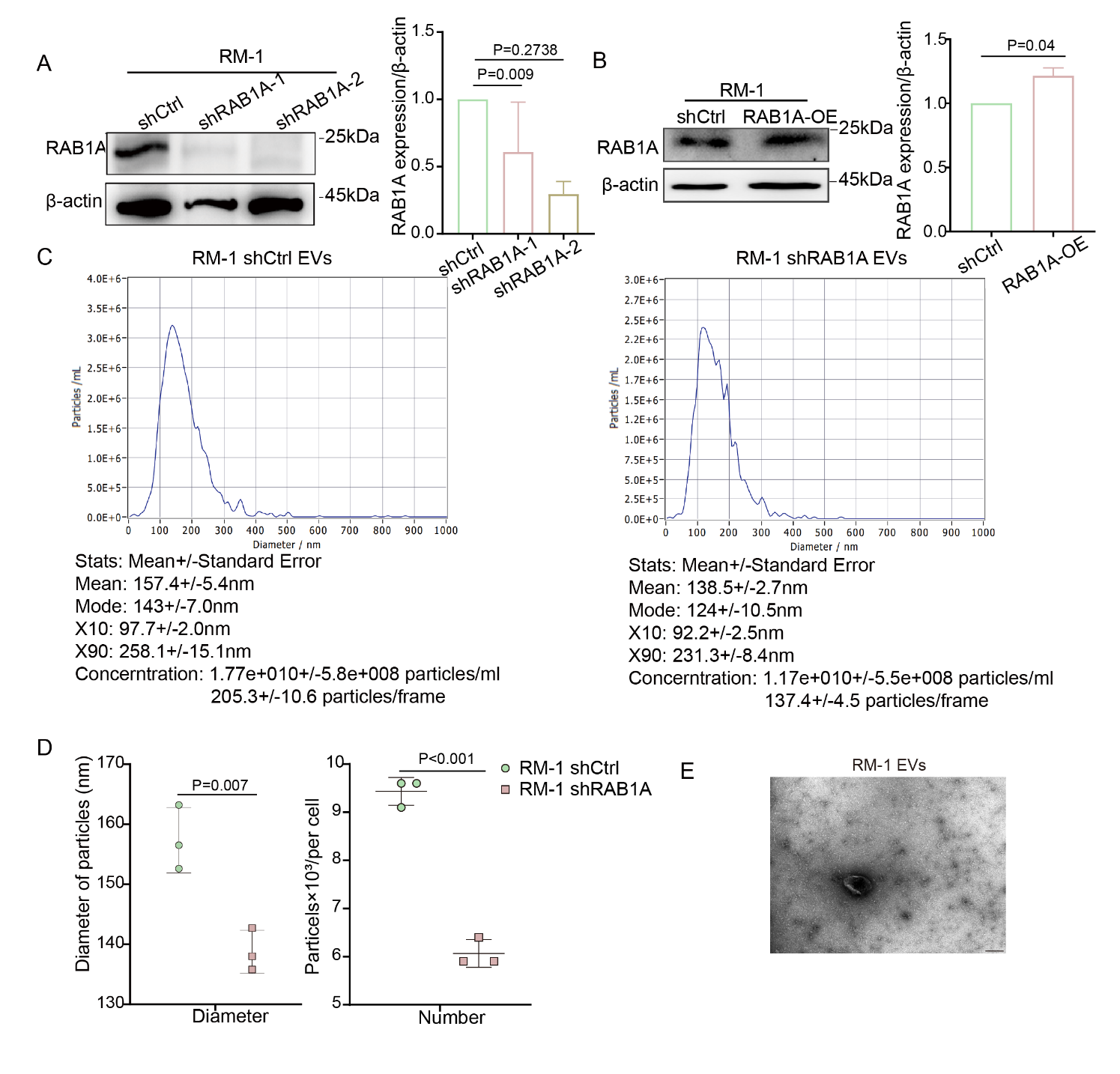


**Supplementary Figure 3.** RAB1A affects EV secretion in RM-1 cells. (A) Knockdown efficiency of RAB1A in RM-1 cells. (B) Statistical analysis of RAB1A knockdown efficiency. (C) NTA results of EVs secreted by shCtrl and shRAB1A RM-1 cells. (D) Statistical analysis of NTA. (E) Representative TEM images of EVs (scale bar: 100 nm). All experiments have been repeated at least three times. Data were analyzed using t-test. P < 0.05 was considered statistically significant.


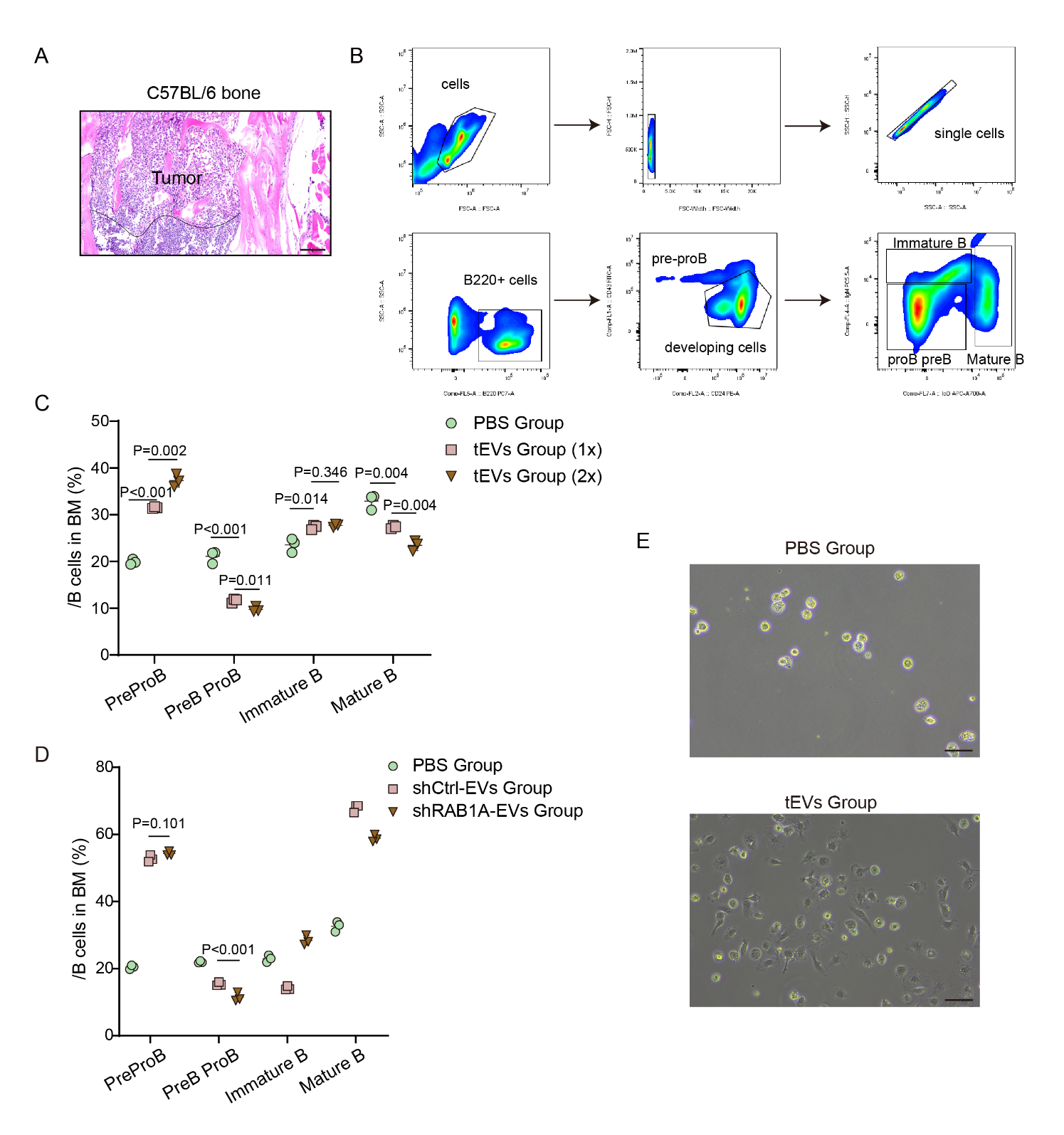


**Supplementary Figure 4.** Both tEVs and murine PCa cells dysregulate the proportions of lymphocytes and myeloid cells. (A) Representative H&E staining of bone from C57BL/6 mice with bone metastasis (scale bar: 50 µm). (B) Representative flow cytometric analysis of B subtypes in bone marrow. (C) Dose-dependent effects of tEVs on B cell subpopulations in cultured bone marrow cells (1×, 5 × 108 particles/well; 2×, 1 × 109 particles/well). (D) Quantified percentages of B cell subtypes in bone marrow cells cultured with shCtrl-EVs or shRAB1A-EVs. (E) Representative imaging of bone marrow cells after tEV treatment (scale bar: 20 µm). All experiments have been repeated at least three times. Data were analyzed using one-way ANOVA with multiple comparisons test (C-D). P < 0.05 was considered statistically significant.


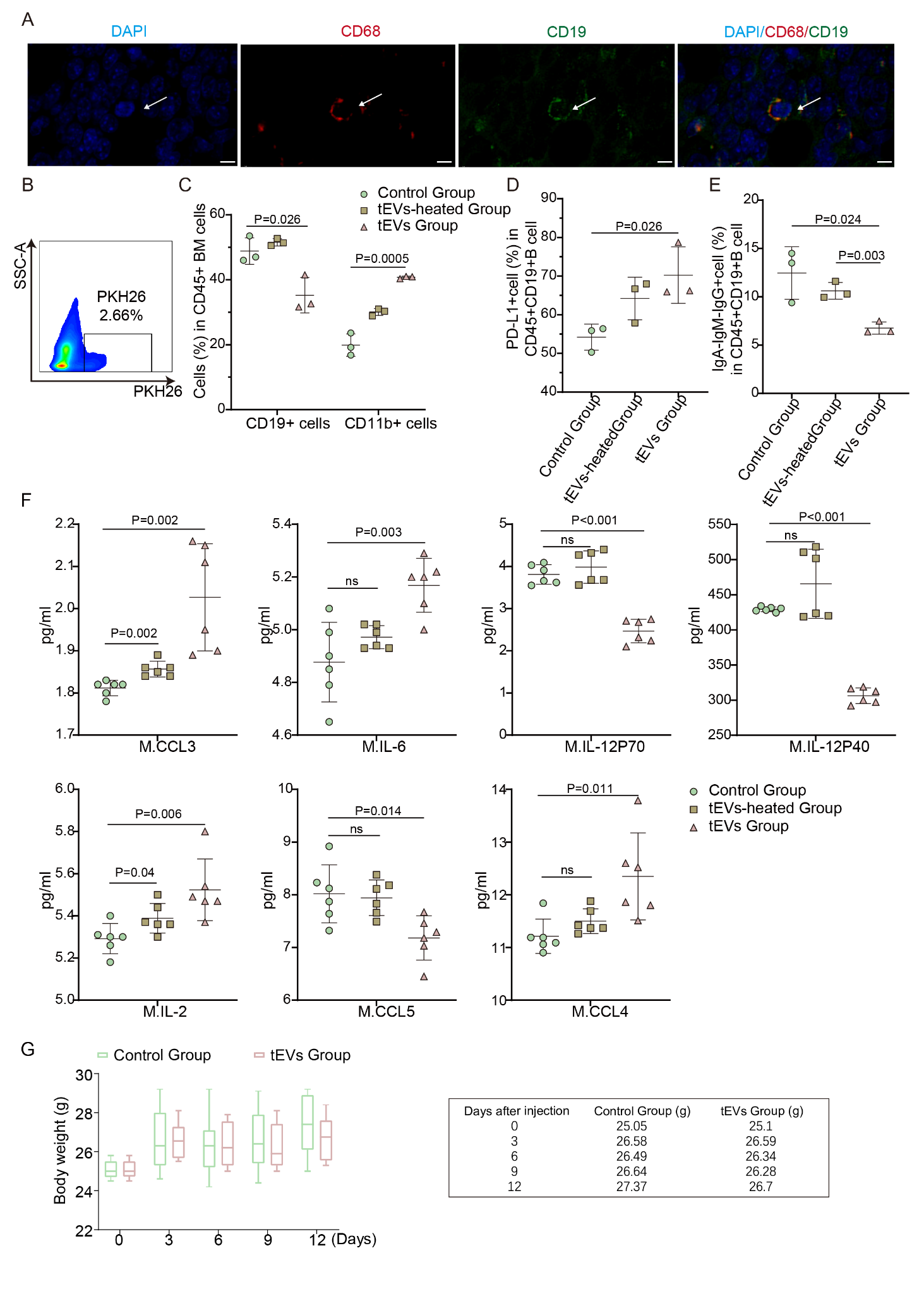


**Supplementary Figure 5.** EVs derived from PCa influence immune microenvironment. (A) IF imaging of tumor sections stained for CD68 (red), CD19 (green), and DAPI (blue) in PCa model with bone metastasis (scale bar: 200 µm). (B) Analysis of bone marrow cells by flow cytometry 4 hours after intravenous injection of PKH26-labeled tEVs. (C) Frequencies of B220+ B cells and CD11b+ myeloid cells in PB of mice (n=3). (D) Frequency of PD-L1⁺ B cells among CD45⁺CD19⁺ B cells in PB (n=3). (E) Proportion of IgA⁻IgM⁻IgG⁺ B cells among CD45⁺CD19⁺ B cells in PB (n=3). (F) ELISA assay performed on mouse plasma samples (n=3). (G) Body weight of mice. All experiments have been repeated at least three times. Data were analyzed using t-test and one-way ANOVA with multiple comparisons test (A). P < 0.05 was considered statistically significant.


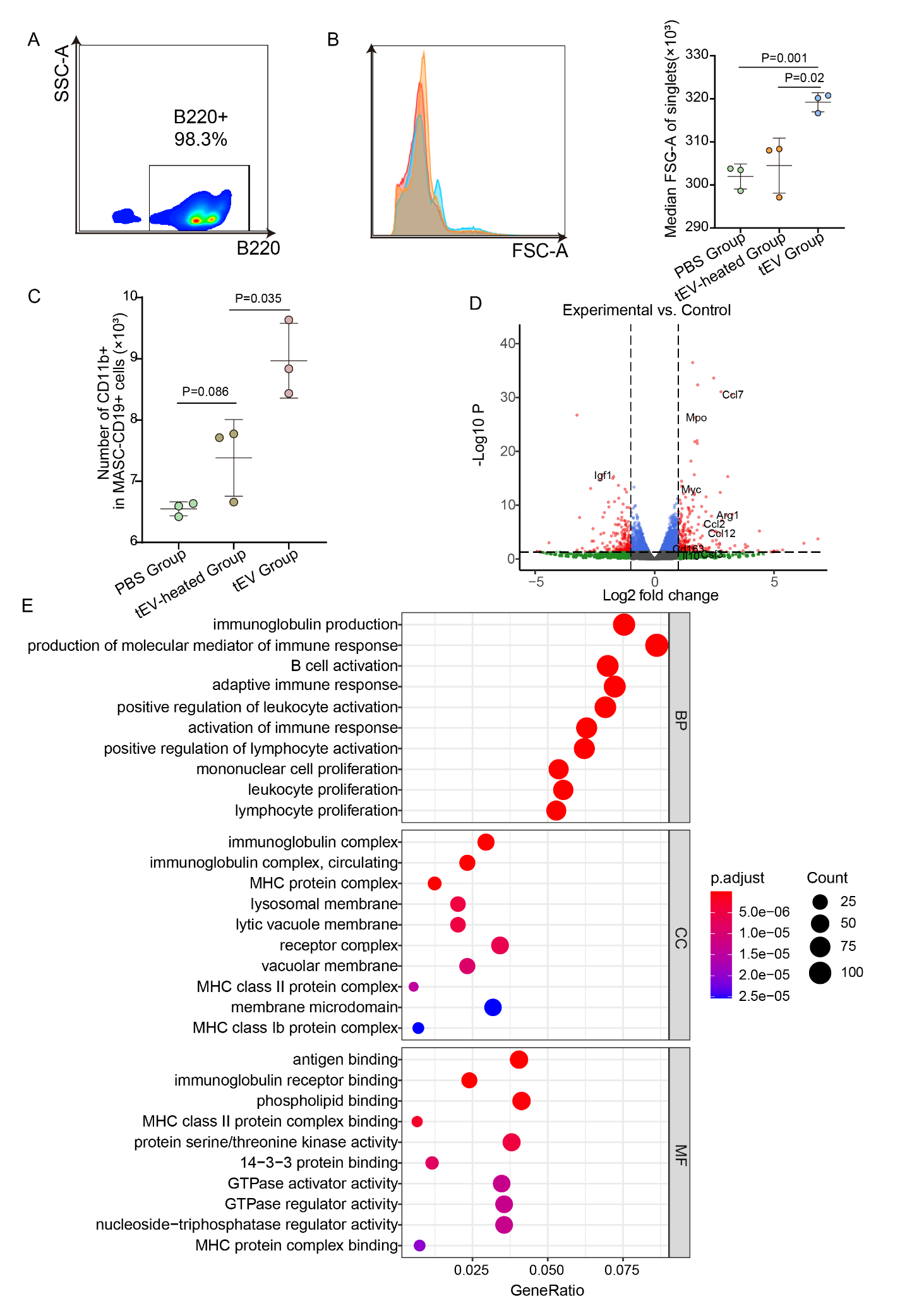


**Supplementary Figure 6.** EVs impair the development of MACS-CD19⁺ B cells in vitro. (A) Purity of MACS-purified CD19⁺ B cells. (B) Median FSC‑A of CD19+ cell population in different groups. (C) Total count of myeloid cells (B220-CD11b⁺) calculated by flow cytometry in 1×106 CD19⁺ cells treated with tEVs. (D) Volcano plot of DEGs. (E) GO enrichment analysis of DEGs downregulated in CD19⁺ cells treated with tEVs compared to control cells. All experiments have been repeated at least three times. Data were analyzed using one-way ANOVA with multiple comparisons test (B-C). P < 0.05 was considered statistically significant.


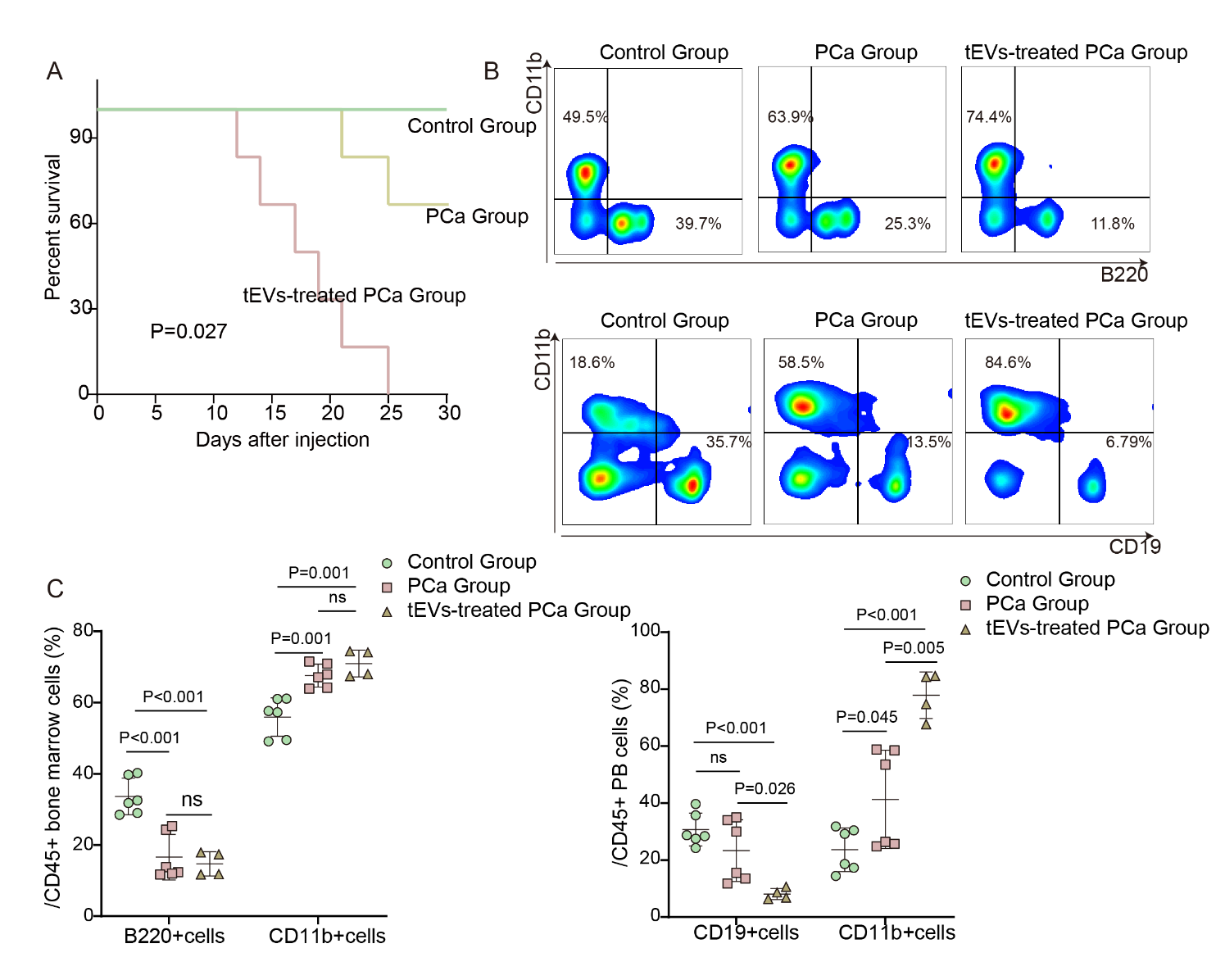


**Supplementary Figure 7.** Pretreatment with tEVs shortens survival time and disrupts cell subtypes in mice. (A) Kaplan-Meier survival analysis of experimental mice. (B) Gating strategy for B cells and myeloid cells in bone marrow (top) and peripheral blood (bottom). (C) Quantification of B cells and myeloid cells in BM (left) and PB (right). Control Group, n=6; PCa Group, n=6; tEVs-treated PCa Group, n=4. All experiments have been repeated at least three times. Data were analyzed using one-way ANOVA with multiple comparisons test (B). P < 0.05 was considered statistically significant.

## Supplementary Tables

**Supplementary Table 1.** Primers for mRNA expression analysis.

**Supplementary Table 2.** Flow antibodies.

**Supplementary Table 3.** DEGs between experimental groups and control groups.
